# Advances and trends in meningioma research over the last decade: A scientometric and visual analysis

**DOI:** 10.3389/fonc.2023.1112018

**Published:** 2023-03-08

**Authors:** Tingbao Zhang, Yu Feng, Kui Liu, Zheng Liu

**Affiliations:** Department of Neurosurgery, Zhongnan Hospital of Wuhan University, Wuhan, China

**Keywords:** meningioma, scientometric, treatment, classification, molecular characteristics

## Abstract

**Objective:**

We conducted a scientometric and visual analysis of meningioma studies in the past ten years and discussed the current status and trends of meningioma research to provide a reference basis for conducting relevant clinical practice or research.

**Method:**

A search of the topic of meningioma in the Web of Science Core Collection database was conducted for January 2012-December 2021. The scientometric tools CiteSpace (version 5.8.R3), VOS viewer (version 1.6.17), and the Bibliometrix package of R software (version 4.2.1) were used to visualize and analyze the country of publication, institution, author, keywords, and cited literature of meningioma.

**Results:**

A total of 10,397 documents related to meningioma were collected, of which 6,714 articles were analyzed. The annual analysis shows an increase in published articles, with an annual growth rate of 8.9%. 26,696 authors from 111 countries or regions were involved in publishing relevant studies. The country with the highest number of publications was the United States (1671), and the institution with the highest number of publications was the University of California, San Francisco (242). The keyword clustering of current studies can be grouped into five groups: meningioma characteristics and basic research, surgical treatment, radiation therapy, stereotactic radiosurgery, and management of complications. Keyword trend analysis shows that meningioma classification and molecular characteristics are emerging hotspots for meningioma research in recent years.

**Conclusion:**

The scientometric and visual analysis demonstrated the research status and trends of meningioma. Over the past decade, meningioma research has focused on managing meningiomas with a predominance of surgical treatment and radiation therapy. At the same time, meningioma classification and molecular characteristics are emerging as current and possible research hotspots in the coming period.

## Introduction

Meningioma is one of the most common central nervous system tumors, accounting for more than 30% of primary intracranial tumors in adults, second only to glioma, and relatively rare in children and adolescents (0.4% to 4.6%) (1, 2). Meningiomas originate from the arachnoid cap cells in the dura mater’s inner layer and grow more slowly; most of them are grade WHO I tumors (2, 3). Meningioma management is primarily surgical resection and most patients with gross total resection have a good prognosis (4). As a result, meningioma research has not received enough attention in the past compared to more malignant gliomas. Until the last decade, there has been a growing interest in the study of meningiomas, as evidenced by many studies and review articles on the subject. For instance, many attractive new therapeutic targets have been identified in the last decade (5). Various anti-angiogenic drugs, genomic-targeted drugs, and immunotherapies have performed exceptionally well in early trials (6, 7). However, a comprehensive scientometric review of the latest research on meningiomas is lacking. There are a few previous scientometric articles on meningioma, including an analysis of the top 100 most cited papers (8) and an analysis of stereotactic radiotherapy for meningioma (9). Although these studies provide a preliminary understanding of meningioma research, a more comprehensive scientometric analysis of meningiomas is not available in the literature.

The scientometric analysis is an emerging tool to quickly explore the structure and trends of a topic or domain through statistical methods and visualization (10–12). It can extract useful information from a large amount of literature by identifying relevant nodes. Currently, commonly used scientometric software includes CiteSpace (13), VOS viewer (14), bibliometrix package of R software (15), Science of Science (SCI2) and HistCite, etc. (16). Among them, CiteSpace and VOS viewer are the most popular ones due to their convenience and authority. CiteSpace is a scientometric software developed by Professor Chaomei Chen, a leading informatics expert at Drexel University, based on citation analysis theory and using the Java language (17). It enables researchers to find the most relevant topics and scientific literature in their field of knowledge and to understand the most critical valid information. Moreover, it clarifies the field’s development process and identifies current research frontiers and trends. VOS viewer is a software application for visual analysis of scientific literature developed by Leiden University in the Netherlands to create, visualize and explore information maps based on web data (18). VOS viewer is based on a clustering analysis algorithm to realize scientific knowledge mapping, showing the structure, evolution, cooperation and other relationships in the knowledge domain. Moreover, its outstanding feature is its graphic solid display capability and suitability for large-scale data.

In this study, we used CiteSpace and VOS viewer in combination with the bibliometrix package of R software for scientometric and visual analysis of meningioma studies in the past 10 years. Meanwhile, we used artificial statistical screening to analyze keywords for meningioma classification, treatment, and molecular characteristics based on scientometric analysis. The combination of scientometric analysis and historical review will identify key evidence and highlight emerging meningioma research trends.

## Materials and methods

**Figure 1:**
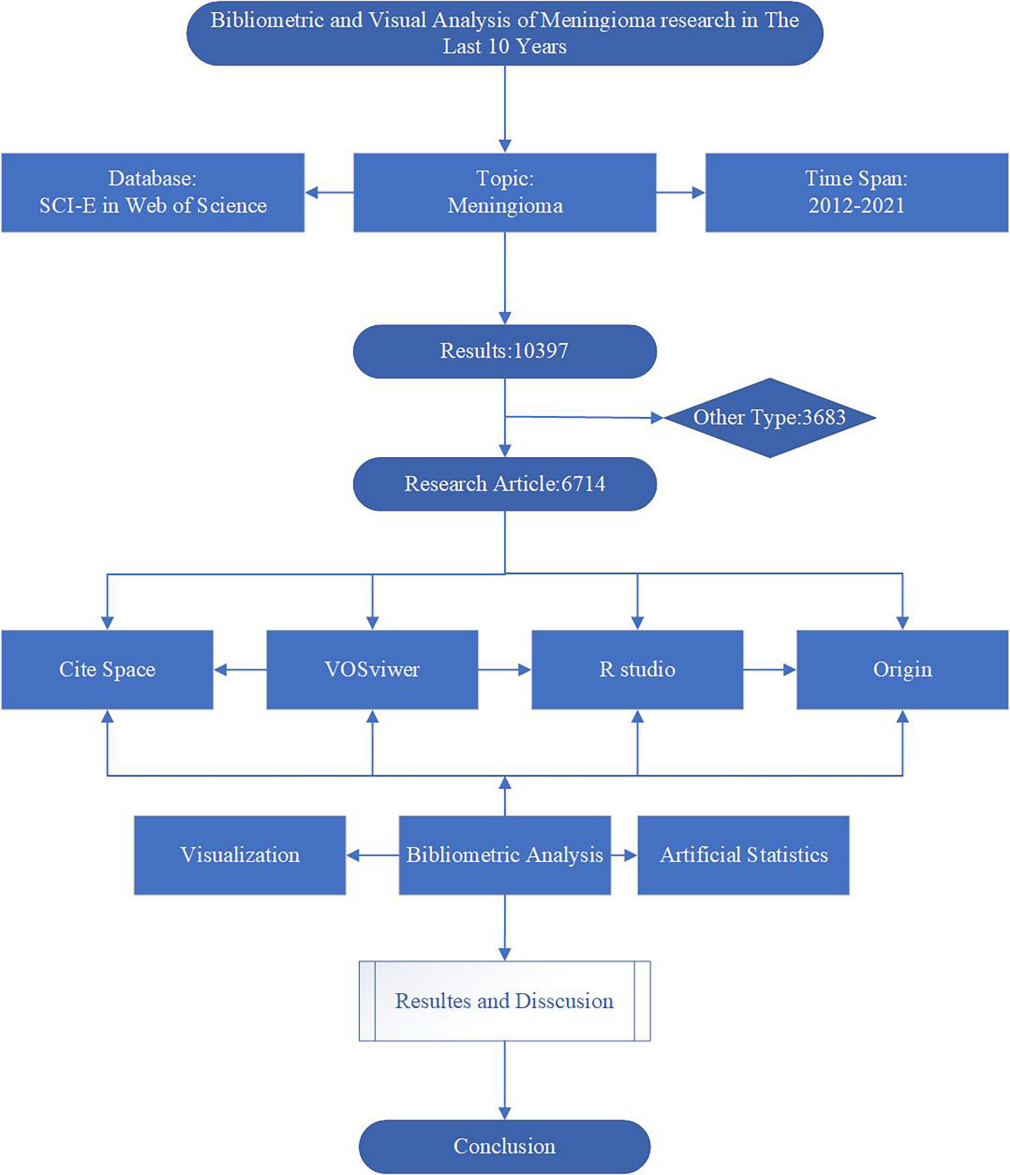
Flowchart of Scientometric and Visual Analytics.

Given that the Web of Science Core Collection (WoSCC), the most commonly used database for scientific or scientometric analysis, contains all the essential information used for the analysis, we chose WOS as our data source (19). We use WoSCC as our data source. All data were retrieved from WoSCC on October 01, 2022, to avoid possible bias due to continuous database updates. We used “meningioma*” as a subject search term and set the period from 2012 to 2021, limiting the type of literature to articles. The detailed search and analysis process is shown in **Figure 1**.

First, the collected data were scientometric analyzed using the bibliometrix package of R software (version 4.2.1), including overall characteristics, annual publications, topographic maps of national collaborations, and trend maps of authors and keywords. Combined with the scientometric results, bar charts of publications and citations by country/region, institutions, authors and journals were created using Origin 2022. Also, the H/G/M index was added to the journal bar chart to analyze the scientometric results on journal impact comprehensively. Then, a network graph of country collaboration, a keyword co-citation trend graph and a cluster analysis graph were visually analyzed using VOS viewer (version 1.6.17). Different colors indicate the clusters in the graph, and the collaboration or co-citation connecting lines are indicated. The size of the circles indicates the number of documents, references or keywords. Finally, a literature citation node analysis was performed using CiteSpace (version 5.8. R3).to find the most cited keywords. The parameter settings included time slices (2012-2021) and selection criteria (cited more than 50). In addition, we performed artificial statistical analysis of keywords based on scientometric results to filter out the top 10 most cited keywords in different directions and visualized and analyzed them using Origin 2022.

## Results

### General characteristics and annual analysis

**Figure 2:**
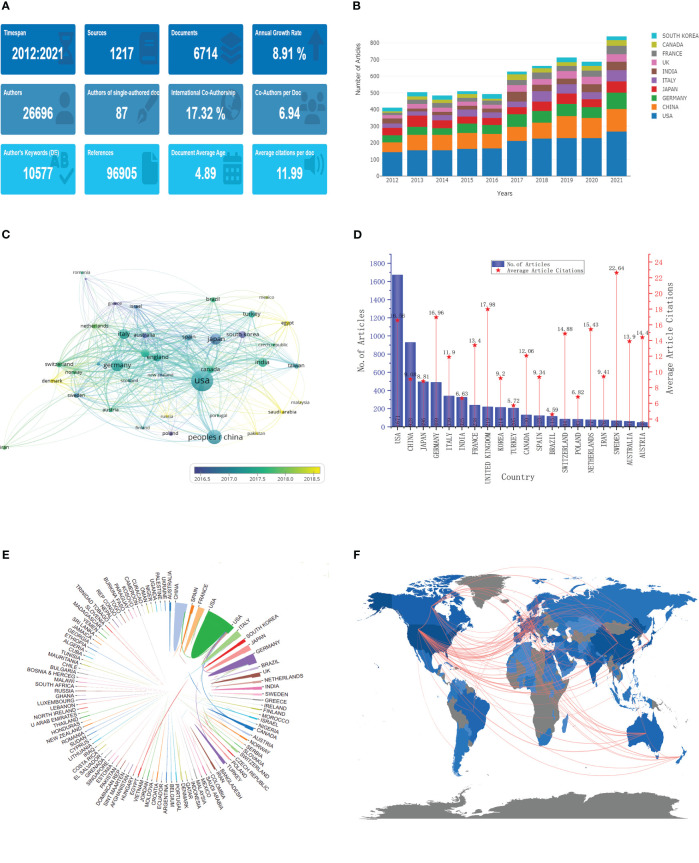
General characteristics and annual, country and cooperation analysis. **(A)** General characteristics; **(B)** Annual analysis; **(C)** Publication volume and trends in various countries; **(D)** Number of articles published and average number of articles cited in various countries; **(E, F)**. Cooperation between the various countries.

From 2012 to 2021, 10,397 documents were published on “meningioma”, including 6,714 articles (**Figure 2A**). These articles were published in 1217 journals by 26696 authors, with an average of 6.94 co-authors per article; 87 of these articles were independently authored, and another 17.32% were published in international collaboration. 96,905 references were cited in these articles, with an average of 11.99 citations per article. In addition, these articles have been cited 80,501 times, with an average of 11.99 citations per article.

The annual number of publications in the last decade tended to increase each year, especially from 2016 to 2017 (**Figure 2B**); the annual number of publications increased nearly 1-fold from 483 in 2012 to 935 in 2021, with an average annual growth rate of 8.91%. Each country’s annual publication volume also shows a yearly performance increase. The United States has the highest number of publications (1671), with more than 1000, followed by China with 928, Japan with 496, Germany with 489, and Italy with 339 (**Figure 2C**). Some countries, such as South Korea and France, have concentrated their publications in the early years. In contrast, others have concentrated in recent years, such as Denmark, Egypt, and Saudi Arabia.

### Analysis of the influence of countries/regions and cooperation

A total of 111 countries/regions were involved in meningioma-related research, and their differences in impact were related to the volume of articles published (**Figure 2D**). The overall citation volume of country articles was ranked the same as the volume of publications, with the top five being the United States (27674), China (8427), Germany (8293), Japan (4368), and Italy (4033). However, the number of citations of a single article in different countries shows varying levels. Switzerland has the highest number of citations for a single article, with 22.64, followed by the UK with 17.96 and Germany with 18.86. Although the US and China rank first and second in total citations, they rank 4th and 14th in citations for a single article, with 16.56 and 9.08. The articles published in collaboration between different institutions are mainly domestic but also partially (17.32%) international (**Figures 2E, F**). The most significant inter-country collaborations were in the United States, with China, Germany, and Canada as the leading collaboration countries.

### Analysis of institutions, authors, and journals

**Figure 3:**
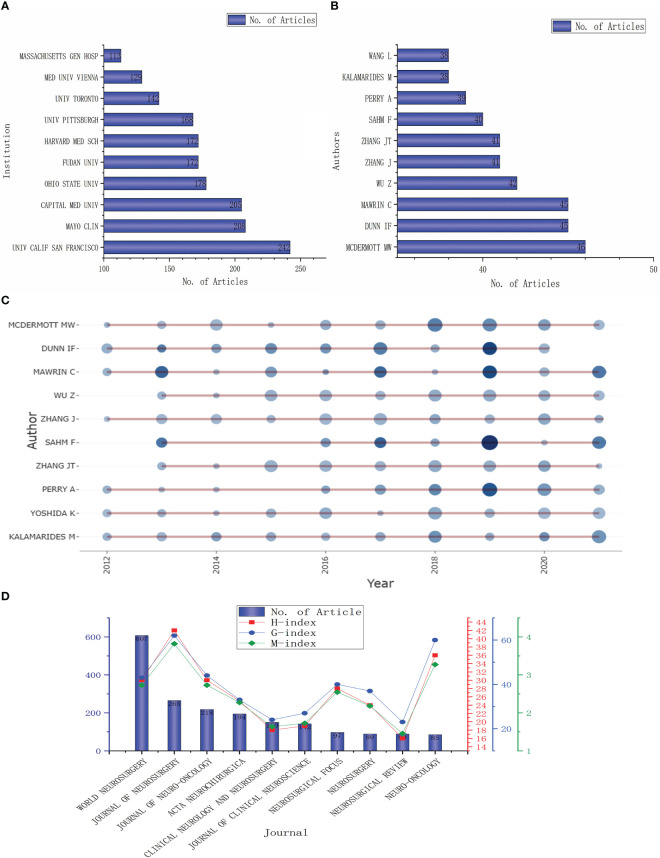
Analysis of institutions, authors and journals. **(A, B)**. The top 10 institutions and authors by published volume; **(C)** The time distribution of the first ten authors’ published volume; **(D)** The number of articles published in the top 10 journals and their influence.

These articles originated from 4835 institutions, with the top 10 publishing more than 100 articles (**Figure 3A**). Three of the top five institutions are from the United States: The University of California (242 articles), Mayo Clinic (208 articles), and Ohio State University (178 articles); the other two institutions are from China: Capital Medical University (205 articles) and Fudan University (172 articles). The top 10 authors are shown in **Figure 3B**, mainly from China (4), the United States (3), and Germany (2), with the highest number of publications coming from WM (46), followed by DF from the United States and MC from Germany (45). The distribution of the top 20 authors in terms of publication volume is shown in **Figure 3C**. From the figure, it can be seen that these authors have published at least one article almost every year in the last ten years, with the highest number of articles published in 2019.

The top 10 journals with the most articles related to meningioma are listed in **Figure 3D**. The most published journal is WORLD NEUROSURGERY (607 articles), accounting for about one-tenth of the total number of articles, followed by the JOURNAL OF NEUROSURGERY (265 articles) and JOURNAL OF NEURO-ONCOLOGY (218 articles). The journals with more than 100 articles also include ACTA NEUROCHIRURGICA (194 articles), CLINICAL NEUROLOGY AND NEUROSURGERY (150 articles), and JOURNAL OF CLINICAL NEUROSCIENCE (142 articles). However, the impact of these journals is not proportional to their number of publications. H-index, g-index, and m-index are the highest for JOURNAL OF NEUROSURGERY with 42, 62, and 3.818, respectively. Followed by NEURO-ONCOLOGY (36, 60, and 3.273)

### Keyword clustering and trending analysis

**Figure 4:**
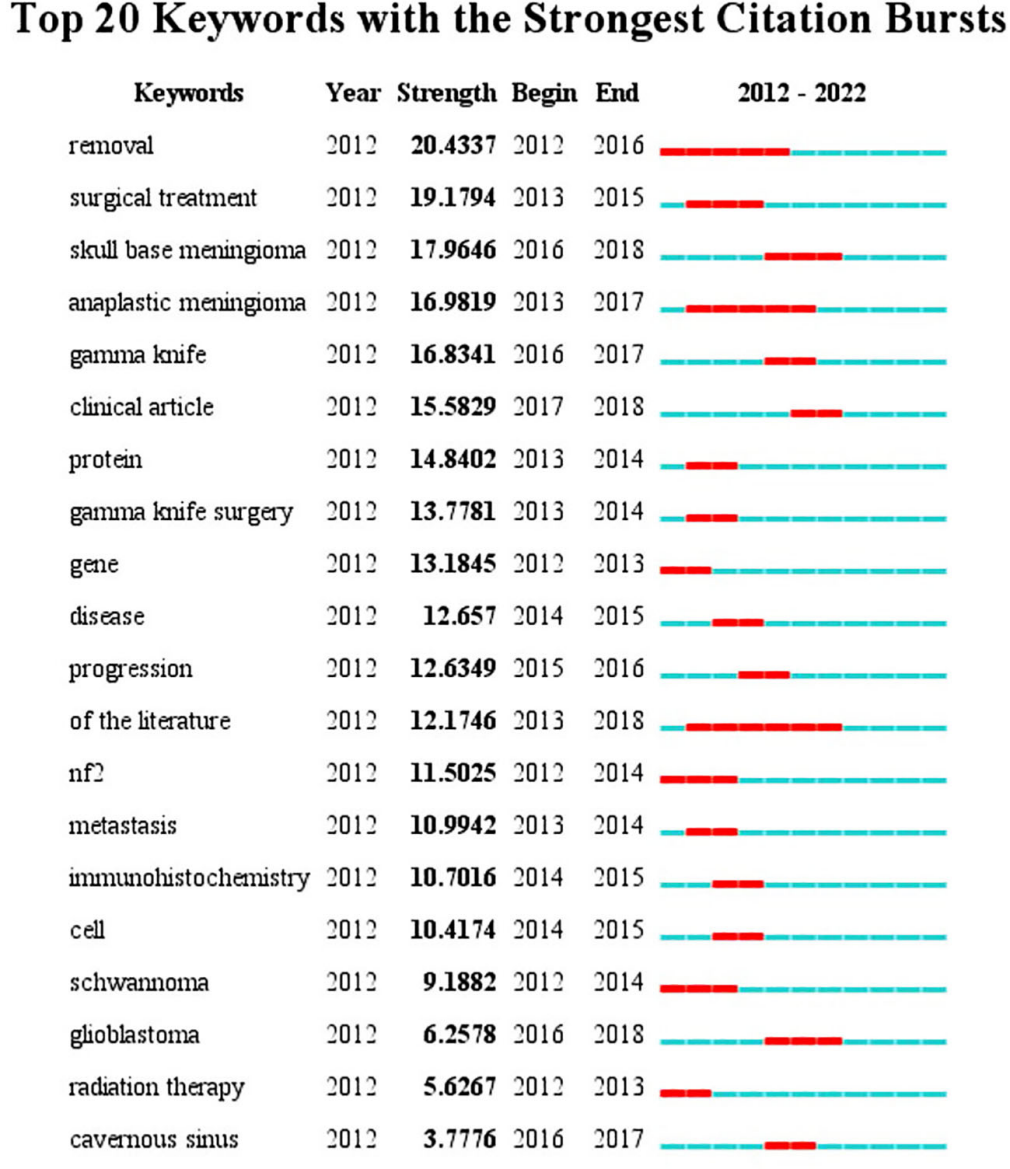
Top 20 keywords with the strongest citation bursts.

**Figure 5:**
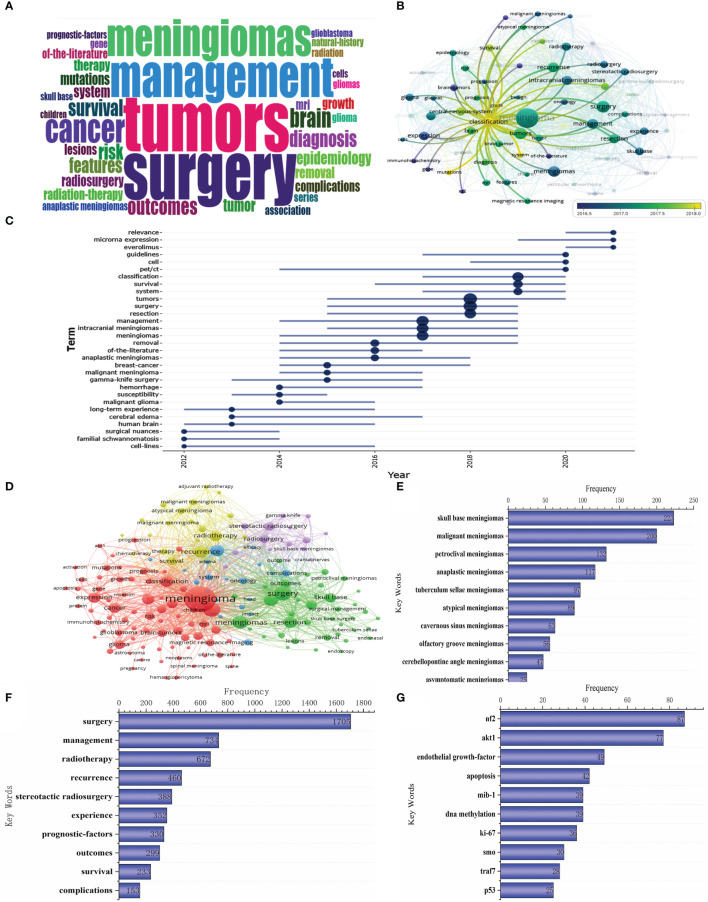
Keyword clustering, trend analysis and manual analysis. **(A)** Keyword Cloud; **(B, C)** Keyword trend analysis; **(D)** Keyword clustering analysis; **(E)** Keywords related to meningioma classification; **(F)** Keywords related to meningioma treatment; **(G)** Keywords related to meningioma molecular characteristics.

The top 20 most frequently used keywords in this literature are shown in **Figure 4**. As can be seen from the chart, the two most used keywords are related to surgical resection, which are “resection” and “surgical treatment”; the top 5 keywords are also “skull base meningioma”, “mesenchymal meningioma”, and “gamma knife”. Regarding period, the three keywords with the largest span were “resection”, “mesenchymal meningioma”, and “literature”. The keywords at the time of WOS inclusion were displayed according to the utilization size (see **Figure 5A**). From the word cloud, it can be seen that the top 5 most recorded keywords are: “tumor”, “surgery”, “management”, “meningioma”, and “cancer”, in that order. VOS viewer was used for co-occurring keywords, and it was found that “classification” became a prominent new keyword around 2018 (**Figure 5B**). A temporal distribution of keywords using citations shows that keywords related to tumor surgery, such as “surgery”, “resection”, and “management”, have been used (**Figure 5C**). In addition, it can be seen that the focus keywords used in the last 3 years, such as “cell”, “microRNA expression”, and “ewelmer”, are related to basic research and molecular characteristics of tumors. It indicates that the research hotspots of meningioma in recent years have gradually favored basic research.

At the same time, we also performed cluster analysis on the keywords. The keywords with more than 50 citations were clustered using VOS viewer (**Figure 5D**). We could see that these keywords were clustered into 5 categories: (i) the red part with “meningioma” as the main keyword was mainly related to the general characteristics and basic research of meningioma; (ii)the green part with “surgery” as the main keyword was mainly related to the surgical resection treatment of meningioma; (iii)the yellow part with “recurrence” and “radiotherapy” as the primary keywords is mainly related to the radiotherapy treatment of meningioma. (iv)the purple part with “stereotactic radiosurgery” as the main keyword is mainly related to stereotactic radiosurgery for meningioma; (v)the blue part with “intracranial meningioma” as the main keyword is mainly related to brain edema and other related complications.

In addition, we listed all keywords with more than 20 citations and performed the artificial statistical analysis. First, considering that “classification” became the most used keyword in 2018, we screened the keywords related to meningioma classification (**Figure 5E**). The figure shows that the main classifications include those based on tumor site and pathological type. Secondly, the keywords related to meningioma management were screened and summarized, and the first 10 keywords in the list are shown in **Figure 5F**. As we can see from the figure, the primary treatment modalities include surgical resection and radiotherapy or stereotactic radiosurgery. The main content of the study is about survival after treatment and its impact factors. Finally, considering that meningioma molecular characteristics research has gradually become a hot spot in meningioma research in the past 3 years, we screened the related keywords (**Figure 5G**). It can be seen that the hot molecules in basic meningioma research include nf2, akt1, and endothelial growth factor.

## Discussion

### Major findings

From the results of the scientometric analysis of the last 10 years in this paper, it can be seen that: (i) in recent years, meningioma-related research has increased annually and received attention from various countries, with close cooperation among the countries or regions involved in the research; (ii) some scholars and journals have continued to focus on meningioma-related research and achieve specific results and influence, but the influence is not entirely consistent with the total number of articles issued; (iii) in the last decade, the focus of related studies has been gradually refined, with a shift from studies related to meningioma management with surgical resection and radiation therapy as the theme to a shift focusing on tumor differentiation and molecular characteristics studies. In the following, we discuss three aspects of meningioma classification, treatment, and molecular characteristics, combining the results of this study with related literature.

### Meningioma classification

We can see from the scientometric results that in the keyword trend analysis, “classification” was widely cited as a keyword in the period centered on 2018. We believe this may be related to the publication of the fourth edition of the WHO classification of central nervous system tumors in 2016 (3). In this version of the classification, the diagnostic terminology of “integration” with histological and molecular information has been introduced to improve the accuracy of diagnosis and patient treatment, which is an essential guideline for the clinical diagnosis and treatment of meningioma (20, 21). In the recent 2021 update of the WHO Classification of CNS Tumors, the applicability of pathologic histologic features to the staging of meningiomas was continued, while the importance of biological markers for the classification of different grades of meningiomas was emphasized (2). Therefore, in our keyword trend analysis, we can see that meningioma-related molecular characteristics have become a hot research topic in recent years (see “ Meningioma Related Molecular Characteristics” below).

We conducted an artificial screening and statistical analysis of keywords related to meningioma classification. The results showed that these keywords were mainly divided into two categories. One of them is the anatomical classification, including skull base meningioma (22), petroclival meningioma (23, 24), tuberculum sellae meningioma (25), cavernous sinus meningioma (26), olfactory groove meningioma (27) and cerebellopontine keratoma (28), etc. The surgical approach and operative details of meningiomas at different anatomic sites are also different. These clinical studies mainly discuss the most suitable surgical approach and operation for meningiomas in a particular anatomic site to obtain better surgical prognosis for patients (29). The other classification relates mainly to pathological features, including malignant meningioma (30), atypical meningioma (31), anaplastic meningioma (32), and asymptomatic meningioma (33), etc. This classification is primarily associated with treatment modalities and prognosis, such as atypical and anaplastic meningiomas, which tend to have a poor prognosis and require postoperative adjuvant therapy (34, 35).

### Meningioma treatment

The latest guidelines suggest that asymptomatic meningiomas without occupying effects can be awaited by annual magnetic resonance imaging (MRI) (4). However, growing or symptomatic meningiomas with occupying effects should be treated by maximum safe resection. Moreover, asymptomatic meningiomas managed by observation usually show rapid growth, requiring a shift in management to surgical resection to reduce the occupancy effect. Most patients have a favorable outcome with maximal resection to reduce the occupancy effect. However, the possibility of recurrence exists for patients with incomplete resection or high-grade meningiomas. And the higher the grade of meningioma, with higher recurrence rates and worse survival rates (36). For instance, compared to benign meningiomas, the 5-year recurrence rate of total tumor excision for atypical meningiomas is 35% to 38%, and the risk of recurrence is 7 to 8 times higher than that of benign meningiomas (33). Therefore, postoperative adjuvant radiotherapy or stereotactic radiosurgery should be considered for this group of patients (34, 37).

As seen in the scientometric results of this study, meningioma management occupies the most significant portion of meningioma research in the last decade (38). Most studies related to meningioma resection include surgery details, the extent of resection, management of postoperative complications, and prognostic factors influencing prognosis. Secondly, radiation therapy and stereotactic radiosurgery are the other two primary management modalities after surgical meningioma resection. Related studies have included adjuvant therapy for specific types of meningiomas (e.g., atypical meningioma and mesenchymal meningioma) and their outcomes (5). In addition, we identified many studies on novel drug treatments for meningiomas in our artificial screening and statistical analysis of keywords. These drug treatments for meningiomas are usually considered experimental and are used as remedial treatments without further local treatment options (39–41). For example, targeted drugs such as anti-angiogenic drugs are used in the remedial treatment of meningiomas (42).

### Meningioma molecular characteristics

We can see from our analysis of keyword trends that some keywords related to meningioma molecular characteristics are gradually increasing, such as NF2 and AKT1. The trends may be related to the significant progress in research on meningioma molecular characteristics in recent years. Studies have shown that NF2 variants, including shift mutations, allelic inactivation, and missense mutations, could be detected in approximately 60% of meningiomas (2, 43). In addition to NF2, mutations were found in TRAF7, SMO, KLF4, PI3K and AKT1 (44). Non-NF2 variants of meningiomas are more complex and include Hedgehog signaling pathway variants (SMO, SUFU, PRKAR1A, PTCH1/2, etc.), phosphatidylinositol 3-kinase (PI3K) signaling pathway variants (PTEN, AKT1, PIK3CA, PIK3R1, etc.), chromosome remodeling complex variants (SMARCB1, SMARCE1, ARID1A, PBRM1, etc.) and other gene variants (KLF4, BAP1, POLR2A, DMD, etc.) (43).

Some molecular characteristics are associated with histological subtypes of meningioma. For example, TRAF7 and KLF4 mutations are molecular biological markers of secretory meningioma (43), RAF7, POLR2A, and ATK1 mutations are markers of endothelial meningioma (43, 45), SMARCE1 mutations are markers of clear cell meningioma (46), and BAP1 and PBRM1 mutations are markers of rhabdoid and papillary meningioma (47–49). Another part is related to the degree of tumor malignancy, such as the deletion of histone H3 K27me3 expression is closely associated with meningioma recurrence (50–52), TERT promoter mutation and CDKN2A/B pure deletion are molecular biological markers of CNS WHO grade 3 meningioma (53, 54). In addition, changes in DNA methylation levels or expression of specific genes (e.g., NRDG2, MEG3, PDGFR, etc.) are also closely associated with the development of meningiomas (46, 55). Based on DNA methylation characteristics, meningiomas can be subtyped, with differences in anatomic sites, driver genes, and clinical prognosis among subtypes (56). The study of meningioma molecular characteristics is beneficial for further typing of meningiomas and diagnosing and treating tumors. It has been gradually becoming a hot issue in meningioma research.

## Conclusion

A scientometric and visual analysis of meningioma research over the last decade demonstrates its current status and trends to some extent. The main research direction is meningioma management based on surgical resection, radiotherapy, or stereotactic radiosurgery. In recent years, with the progress of basic meningioma research, meningioma classification and molecular characteristics studies have gradually become hot spots for research. In the future, research related to meningioma molecular characteristics may further increase and significantly influence molecular diagnosis and precision treatment of meningioma.

## Author contributions

TZ, KL, and ZL contributed to conception and design of the study. TZ and YF organized the database. TZ performed the statistical analysis. YF wrote the first draft of the manuscript. TZ, YF, KL, and ZL wrote sections of the manuscript. All authors contributed to manuscript revision, read, and approved the submitted version.
